# Generalized Edema and Pseudothrombocytopenia After ChAdOx1 nCoV-19 COVID-19 Vaccination: A Case Report

**DOI:** 10.3389/fpubh.2022.907652

**Published:** 2022-05-27

**Authors:** Joanna Bokel, Daniela P. Mendes-de-Almeida, Remy Martins-Gonçalves, Lohanna Palhinha, Alexandre G. Vizzoni, Danusa Ferreira Correa, Luciana Gomes Pedro Brandão, Patrícia T. Bozza, Beatriz Grinsztejn

**Affiliations:** ^1^Department of Hematology, Instituto Nacional de Infectologia Evandro Chagas, Fundação Oswaldo Cruz (FIOCRUZ), Rio de Janeiro, Brazil; ^2^Onco-Hematology Unit, Clínica São Vicente, Rio de Janeiro, Brazil; ^3^Research Center, Instituto Nacional de Câncer, Rio de Janeiro, Brazil; ^4^Laboratory of Immunopharmacology, Instituto Oswaldo Cruz, Fundação Oswaldo Cruz (FIOCRUZ), Rio de Janeiro, Brazil; ^5^Health Surveillance and Immunization Research Unit, Instituto Nacional de Infectologia Evandro Chagas, Fundação Oswaldo Cruz (FIOCRUZ), Rio de Janeiro, Brazil; ^6^Laboratory of Clinical Research on STD/AIDS, Instituto Nacional de Infectologia Evandro Chagas, Fundação Oswaldo Cruz (FIOCRUZ), Rio de Janeiro, Brazil

**Keywords:** generalized edema, pseudothrombocytopenia, spurious thrombocytopenia, COVID-19 vaccine safety, vaccine-induced pseudothrombocytopenia

## Abstract

Reports of side effects of vaccines against severe acute respiratory syndrome coronavirus 2 (SARS-CoV-2) are increasing worldwide. Capillary leak syndrome and vaccine-induced immune thrombotic thrombocytopenia are very rare but life-threatening adverse events that should be identified early and treated. However, isolated thrombocytopenia can indicate pseudothrombocytopenia. In certain people, ethylenediaminetetraacetic acid (EDTA) induces an *in vitro* platelet aggregation, resulting in misleading underestimation of platelet counts. It is essential to recognize pseudothrombocytopenia to prevent diagnostic errors, overtreatment, anxiety, and unnecessary invasive procedures. We present a case who developed generalized edema and persistent pseudothrombocytopenia after the first dose of the ChAdOx1 nCoV-19 vaccine (AstraZeneca).

## Introduction

Side effects of vaccines against severe acute respiratory syndrome coronavirus 2 (SARS-CoV-2) have been increasingly reported worldwide. Systemic capillary leak syndrome (SCLS), a cause of edema after vaccination, is an extremely rare condition caused by fluid leak from small blood vessels mainly in individuals with a previous history of this syndrome.^1^ Causes of thrombocytopenia following vaccination can have a broad clinical spectrum, ranging from asymptomatic laboratory findings to catastrophic events. Adenovirus vectoral vaccines were associated with vaccine-induced immune thrombotic thrombocytopenia (VITT), a rare but life-threatening adverse event that occurs 5–30 days after vaccination, most commonly after the first dose (1). It is clinically manifested by thrombosis in atypical sites, such as cerebral venous sinus or splanchnic vessels, thrombocytopenia, strikingly high D-dimer levels, and positive anti-PF4 ELISA ^1^ antibodies (2). Once VITT diagnosis is suspected, prompt treatment with immunoglobulin, non-heparin anticoagulation, and in some instances, corticosteroids, plasma exchange, and fibrinogen replacement should be administered (2). However, isolated thrombocytopenia after vaccination can indicate more frequent conditions, such as immune thrombocytopenic purpura (ITP) (3) or pseudothrombocytopenia. Recently, a case was reported of transient pseudothrombocytopenia (VIP) after Ad26.COV2.S vaccination (4).

Platelet counts are usually determined by automated hematologic analyzers using ethylenediaminetetraacetic acid (EDTA)-anticoagulated blood specimens in routine clinical care. In rare cases, EDTA induces time- and temperature-dependent *in vitro* platelet aggregation, resulting in platelet count underestimation known as pseudothrombocytopenia or spurious thrombocytopenia. Although it occurs most often in EDTA–anticoagulated blood, other anticoagulants have also been implicated (5). Mainly due to the presence of EDTA-dependent antiplatelet antibodies, it is a relatively common laboratory finding in thrombocytopenia investigation and can lead to diagnostic errors, overtreatment, anxiety, unnecessary invasive testing, or surgery (splenectomy). It does not encompass platelet number or function abnormalities and can be readily missed if not considered in the differential diagnosis.

Herein we present a clinical case of generalized edema and persistent pseudothrombocytopenia after the first dose of the ChAdOx1 nCoV-19 vaccine (AstraZeneca), with immunophenotypical platelet characterization.

## Case Description

**Figure 1:**
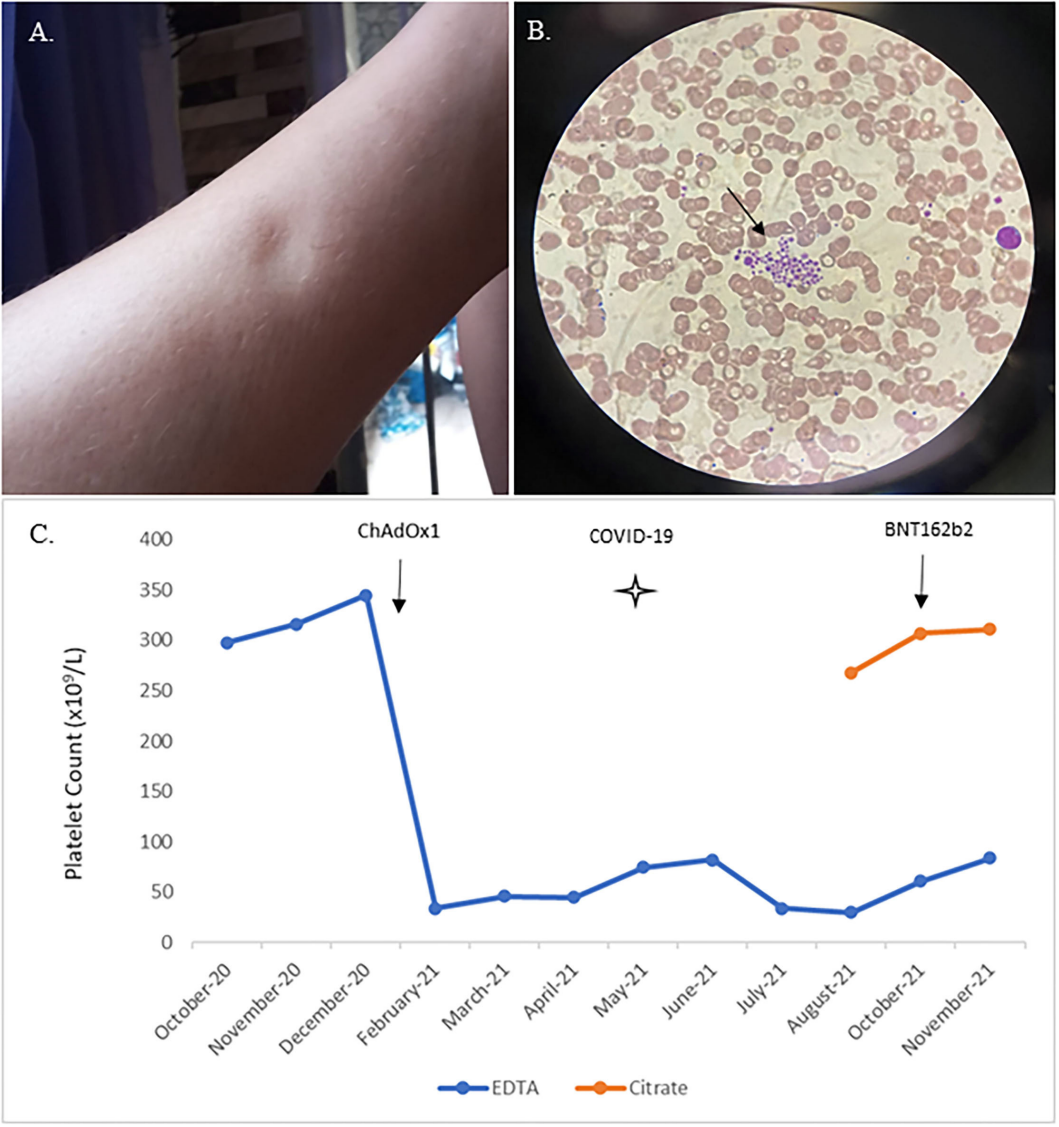
Skin edema, platelet aggregates on the EDTA blood smear, and platelet count evolution in EDTA and citrate anticoagulated blood. Lower limb skin edema in patient 12 days after ChAdOx1 nCoV-19 vaccination, with spontaneous resolution after four months **(A)**; Peripheral blood smear from the EDTA-anticoagulated patient's blood showing platelet aggregates (arrow) **(B)**; Platelet counts in EDTA anticoagulation are shown in blue; platelet counts in citrate anticoagulation are shown in orange starting in August 2021. COVID-19 infection is indicated by a star, ChAdOx1 nCoV-19 and BNT162b2 vaccinations are shown by black arrow s **(C)**.

**Figure 2:**
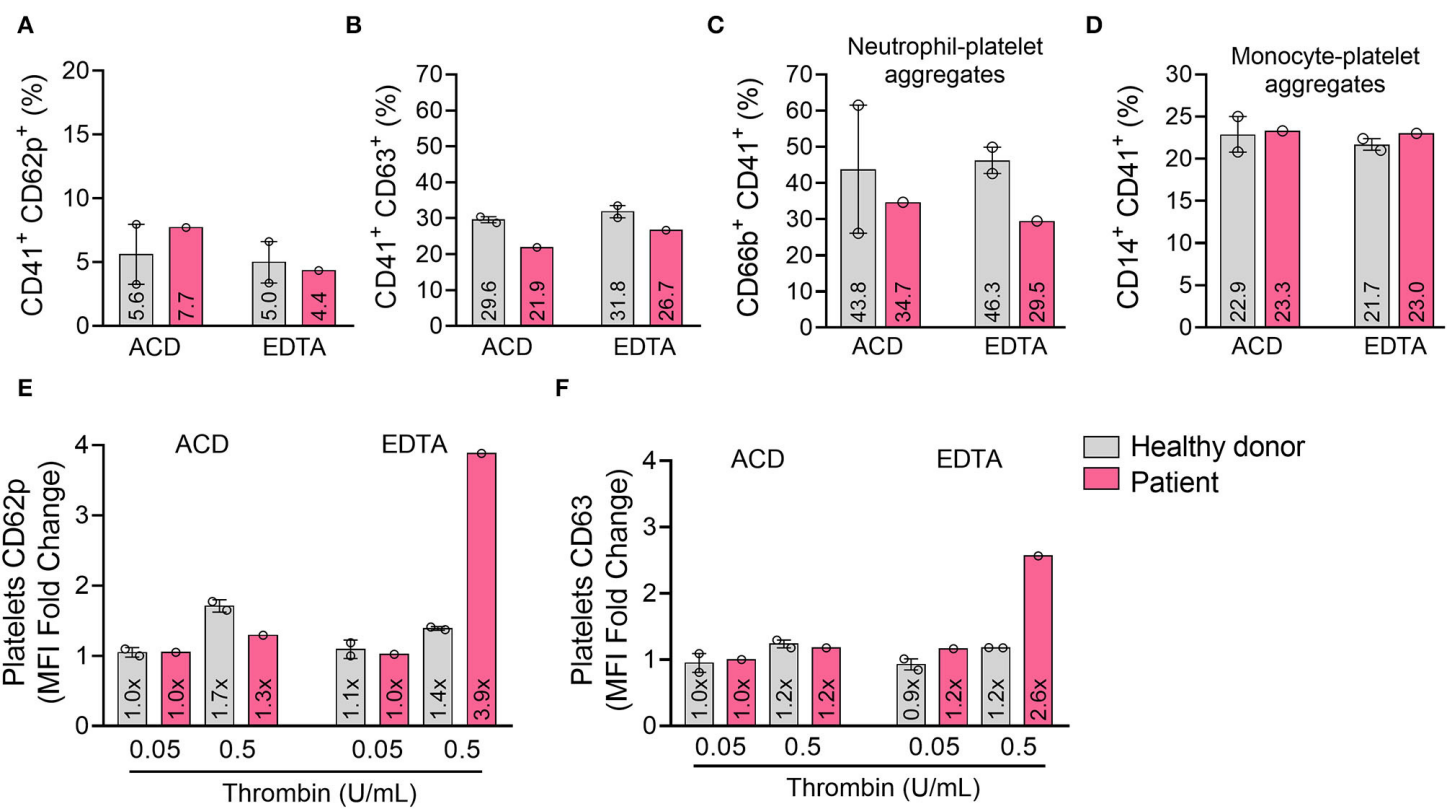
EDTA induced platelets hyperreactivity but did not increase basal activity or heterotypic aggregation with leukocytes. Platelets were isolated from the patient and healthy donors' fresh blood collected with ACD or EDTA and analyzed by flow cytometry for surface expression of CD62p **(A)** and CD63 **(B)**. Platelet-leukocyte aggregates were assessed by the percentage of CD41^+^ platelets among neutrophils (CD66b^+^) **(C)**, total monocytes (CD14^+^) **(D)**, Platelet reactivity was analyzed by the mean fluorescence intensity (MFI) of CD62p **(E)** and CD63 **(F)** after stimulation with thrombin at 0.05 and 0.5 U/mL. Bars represent the mean ± SEM, and each dot represents one individual. ACD: acid-citrate-dextrose.

A 46-years-old white female, smoker with pulmonary emphysema, sought medical consultation in mid-February 2021 after presenting progressive face and lower limbs edema (Figure 1A). She had received the first ChAdOx1 nCoV-19 vaccine dose 12 days before symptoms started, on January 29. She was admitted for investigation in a general hospital named Hospital Duque de Caxias, in Rio de Janeiro. During hospitalization, despite generalized edema, the patient had normal blood pressure (100 × 80 mmHg), no evidence of hemoconcentration (hemoglobin 13.9 g/dL, hematocrit 37.4%), normal liver (total protein 7.5 g/dL, albumin 4.5 g/dL, AST 19 U/L, ALT 18 U/L) and renal functions (creatinine 1.10 mg/dL, urea 33 mg/dL) and her urine sample showed no abnormalities. Arterial and venous doppler of the lower limbs ruled out thrombosis. There was no evidence of rheumatologic disorder (negative antinuclear factor), and her serologies were negative for dengue (IgM), syphilis, HIV, hepatitis C, and hepatitis B infections. She presented an isolated low platelet count (36 × 10^9^/L- reference 150–450 × 10^9^/L) in the automated analyzer, and subsequent counts confirmed this finding. As there was no evidence of bleeding or other life-threatening disorder she was discharged and oriented to continue investigation as an outpatient from the hospital. For comparison, the patient had normal platelet levels documented on a blood count done 1 month before the vaccination in December 2020 and had no blood count done in January 2021. Despite the absence of hemorrhagic symptoms, the assistant physician from the general hospital suspected vaccine-induced ITP and prescribed a course of corticosteroids in March 2021. After 1 month of prednisone 20 mg once a day, she persisted with low platelet count (45 × 10^9^/L platelets), had no significant symptoms, and therapy was discontinued. The edema had progressive spontaneous resolution throughout the investigation, with complete remission after 4 months. In May 2021, she was diagnosed with acute COVID-19 infection and had only mild symptoms, with no need for hospitalization. In July 2021 the patient had no symptoms, maintained the low platelet count and was referenced to Fundação Oswaldo Cruz (Fiocruz/RJ) institute for further investigation. Five months after symptoms onset, she had a consultation with a Fiocruz's hematologist. Blood count using citrate anticoagulant showed normal platelet count, and peripheral blood smear of the EDTA sample showed platelet aggregates (Figure 1B). One month later, the blood counts on EDTA and citrate confirmed these findings (Figure 1C), and pseudothrombocytopenia was diagnosed. She received the BNT162b2 vaccine (Pfizer–BioNTech) as a second dose in October 2021 (9 months after the first one) and reported no symptoms. In November 2021, blood samples from the patient and two age- and sex-matched healthy donors were collected into Acid-Citrate-Dextrose (ACD) and EDTA; platelet activation, hyperreactivity, and aggregation with leukocytes were evaluated through flow cytometry. Both ACD and EDTA samples showed no changes in platelet activation, as demonstrated by CD62p and CD63 surface expression (Figures 2A,B, respectively). Furthermore, no alterations were observed regarding platelet aggregation with neutrophils (Figure 2C) or monocytes (Figure 2D). However, isolated platelets from the patient's EDTA sample, but not ACD, presented hyperreactivity compared to healthy donors, as shown by CD62p and CD63 mean fluorescence intensity (MFI) in platelets stimulated with thrombin (Figures 2E,F). Of note, after centrifugation, the platelets pellet from the patient's EDTA blood sample, but not from healthy donors, was much more challenging to resuspend than the ACD sample, as expected due to increased platelet aggregation with EDTA. Platelet concentrations after resuspension were the same in patients' EDTA and ACD samples (10^9^/mL, data not shown) and in healthy donor samples. The decreased platelet counts in the patient's EDTA samples obtained by automated counting may not account for the enhanced platelet aggregation, thus leading to underestimating platelet counts mistakenly indicating thrombocytopenia. Although it is standard practice to analyze the blood smear and repeat the platelet count using another anticoagulant it was not done in her initial care resulting in a diagnostic failure. Pseudothrombocytopenia was diagnosed only when she was referred to a hematologist specialist, 5 months later. The patient maintained pseudothrombocytopenia when EDTA was used with no clinical findings until February 2022.

## Discussion

The pathophysiology of pseudothrombocytopenia is not clearly defined, but it is suggested that an immune-mediated mechanism accounts for platelet clumping. In the presence of EDTA *in vitro*, cryptic epitopes of the glycoprotein IIb/IIIa complex on the platelet membrane suffer a conformational change and are exposed to acquired or naturally occurring autoantibodies leading to the formation of immune complexes and platelet agglutination. This phenomenon often occurs at low temperatures and has no other laboratory or clinical thrombocytopenia manifestations. Pseudothrombocytopenia could also be related to increased platelet–leukocyte aggregation, characterized by platelet rosetting mainly around monocytes and neutrophils and monocytes, less frequently observed around lymphocytes (6). However, platelet–leukocyte aggregation was ruled out in the blood smear and the flow cytometry analysis of our patient. Alternatively, proteins from alpha granules or thrombospondin expressed on the platelet membrane may cause adhesion to neutrophils and trigger a more generalized agglutination cascade, forming large aggregates. This mechanism is rare and specific for EDTA anticoagulant (6).

EDTA-pseudothrombocytopenia incidence is ~0.07–0.2% in hospitalized patients, increasing to 15.3% in patients investigated for isolated thrombocytopenia (6). Risk factors for pseudothrombocytopenia include hospitalization, males over 50 years old, malignant neoplasms, chronic liver disease, infection, pregnancy, autoimmune diseases, and thrombotic and cardiovascular diseases, heparin-induced thrombocytopenia, surgical settings, post-stem cell transplantation, treatment with valproic acid, insulin, antibiotics, low-molecular-weight heparin, chemotherapeutic agents such as sunitinib, and lately COVID-19 (6–8)^.^ EDTA-pseudothrombocytopenia has also been observed in healthy persons (5).

Pseudothrombocytopenia can typically be identified by collecting information about the patient's history of previous abnormalities on complete blood count or signs and symptoms of platelet disorder; reviewing the peripheral blood smear in the EDTA sample; confirming the finding using a different anticoagulant than EDTA for blood collection, or maintaining the sample at around 37°C before testing (9). Rapid analysis of EDTA blood specimens is advocated to lower the chances of error due to time-dependent falls in platelet counts (6). Because of initial diagnostic failure, our patient experienced iatrogenic immunosuppressive treatment and an immunization delay. As no other cause for the pseudothrombocytopenia was identified, and the EDTA platelet levels became low after ChAdOx1 nCoV-19 vaccine exposure, we hypothesized that this effect was related to the vaccine.

EDTA used as a stabilizer in the ChAdOx1 nCoV-19 vaccine also increases vascular permeability (10) and can cause SCLS. Classical criteria for diagnosing SCLS are diffuse edema, hypoalbuminemia, hemoconcentration, and arterial hypotension. Clinically it can vary from mild symptoms, like edema, to more severe presentations as hypotension and hypovolemic shock (11). The European Medicines Agency safety committee determined that the product information should add SCLS as a vaccine side effect on June 11th 2021 and released a warning to raise awareness among healthcare professionals and patients (12). Despite the edema, our patient did not fulfill the formal criteria for SCLS.

In conclusion, we strongly recommend excluding pseudothrombocytopenia, particularly before further investigation of thrombocytopenia and before starting treatment. We illustrated the first VIP case following a first dose of the COVID-19 ChAdOx1 nCoV-19 vaccine associated with generalized edema. We also presented the longstanding of this diagnosis and the immunophenotypical platelet findings that corroborate the *in vitro* phenomenon. It is essential to perform a comprehensive evaluation of thrombocytopenia following vaccination. Our patient was submitted to an extensive laboratory analysis, missed workdays to attend medical consultations, was put on medical license throughout the diagnostic procedures, had her COVID-19 immunization delayed and was unnecessary medicated with a course of corticosteroids. This falsely low automated platelet count is not associated with a clinical bleeding tendency and does not have any therapeutic consequences, but when misdiagnosed, it can be harmful to the patient.

## Data Availability Statement

The raw data supporting the conclusions of this article will be made available by the authors, without undue reservation.

## Ethics Statement

The studies involving human participants were reviewed and approved by the Review Board of the Evandro Chagas National Institute of Infectious Diseases (#54561321.0.0000.5262). The patients/participants provided their written informed consent to participate in this study.

## Author Contributions

JB, DPMA, PTB, and BG designed the study. JB and DFC attended the patient. DPMA and AGV analyzed the clinical data. RM-G and LGPB performed the platelet assay. JB, DPMA, RM-G, and LP wrote the manuscript. LGPB, BG, and PTB revised the manuscript and supervised the study. All authors have seen and approved the manuscript and its submission.

## Funding

This paper publication was supported by the Fundação de Amparo à Pesquisa do Estado do Rio de Janeiro (FAPERJ).

## Conflict of Interest

The authors declare that the research was conducted in the absence of any commercial or financial relationships that could be construed as a potential conflict of interest.

## Publisher's Note

All claims expressed in this article are solely those of the authors and do not necessarily represent those of their affiliated organizations, or those of the publisher, the editors and the reviewers. Any product that may be evaluated in this article, or claim that may be made by its manufacturer, is not guaranteed or endorsed by the publisher.

## Footnotes

^1^Available online at: https://www.gov.uk/government/publications/coronavirus-covid-19-vaccine-adverse-reactions/coronavirus-vaccine-summary-of-yellow-card-reporting#annex-2-glossary
